# Feature-Based Machine-Learning Spike Marking in Signal and Source Space EEG: A Proof-of-Principle Case Study of Focal Epilepsy

**DOI:** 10.3390/s26196046

**Published:** 2026-09-24

**Authors:** Lala Jafarova, Demet Yeşilbaş, Christoph Kellinghaus, Gabriel Möddel, Stjepana Kovac, Stefan Rampp, Daniela Czernochowski, Sebastian Sager, Ayşegül Güven, Turgay Batbat, Carsten H. Wolters

**Affiliations:** 1 Institute for Biomagnetism and Biosignalanalysis, University of Münster, Malmedyweg 15, 48149 Münster, Germany; l.jafarova@fz-juelich.de (L.J.); demetyesilbass@gmail.com (D.Y.); 2 Department of Cognitive Science, Rheinland-Pfälzische Technische Universität Kaiserslautern-Landau, 67663 Kaiserslautern, Germany; d.czernochowski@rptu.de; 3 Department of Biomedical Engineering, Graduate School of Natural and Applied Sciences, Erciyes University, Kayseri 38039, Turkey; 4 Department of Neurology, Klinikum Osnabrück, Epilepsy Center Münster-Osnabrück, 49076 Osnabrück, Germany; christoph.kellinghaus@klinikum-os.de; 5 Epilepsy Center Münster-Osnabrück, Department of Neurology with Institute of Translational Neurology, University Hospital Münster, 48149 Münster, Germany; gabriel.moeddel@ukmuenster.de (G.M.); stjepana.kovac@ukmuenster.de (S.K.); 6 Department of Neurosurgery, University Hospital Erlangen, 91054 Erlangen, Germany; stefan.rampp@uk-erlangen.de; 7 Department of Mathematics, Otto von Guericke University, 39106 Magdeburg, Germany; sager@ovgu.de; 8 Department of Biomedical Engineering, Faculty of Engineering, Erciyes University, Kayseri 38030, Turkey; aguven@erciyes.edu.tr (A.G.); turgaybatbat@erciyes.edu.tr (T.B.); 9 Otto Creutzfeldt Center for Cognitive and Behavioral Neuroscience, University of Münster, Fliednerstraße 21, 48149 Münster, Germany

**Keywords:** interictal epileptiform discharges, EEG, artificial neural networks, source localization, epilepsy

## Abstract

Accurate detection of interictal epileptiform discharges (IEDs) in electroencephalography (EEG) plays a crucial role in epilepsy diagnosis. Our work investigates the classification of IEDs using Artificial Neural Networks (ANNs) trained on EEG data represented in both signal and source space, in a proof-of-principle case study of focal epilepsy. Source waveforms were computed using a standard clinical source analysis procedure for the dipolar IED topographies, an equivalent current dipole model in a three-compartment head volume conductor model fitted using either a 1-parameter fixed-orientation or a 3-parameter projection approach, both localized to a single best-fit position during the rising flank of the IED. The ANN was trained on raw and feature-extracted versions of signal space and source space data. Feature extraction significantly improved performance across all domains. The highest accuracy (0.98) was achieved in signal space using Katz Fractional Dimension (KFD). In source space analyses, the 1-parameter and 3-parameter models achieved a maximum accuracy of 0.84, with statistical features performing best for the fixed-orientation model and KFD for the free orientation model. Additionally, annotations from three independent expert markers showed considerable variability, with ANN performance falling within the range of inter-expert agreement. While the findings of our proof-of-principle case study support the potential of feature-informed ANN tools to assist expert evaluation in clinical workflows, data from a single patient cannot capture inter-individual variability. Future work on a large, multi-patient cohort is needed to develop a population-generalizable detector.

## 1. Introduction

Epilepsy is a chronic neurological disorder that affects more than 50 million people worldwide. It is characterized by a persistent tendency toward unprovoked, recurrent seizures, caused by abnormal or excessive neuronal activity that leads to changes in behavior, sensation, or motor function [1]. With appropriate diagnosis and treatment, seizure freedom can be achieved in approximately 70% of individuals; however, epilepsy can also impact cognitive, psychological and social functioning [2,3].

Clinical diagnosis begins with the classification of seizures as either focal (originating in one brain area) or generalized (affecting both hemispheres simultaneously), which is essential for selecting appropriate treatment [3,4,5]. Standard diagnostic approaches involve clinical evaluation, electroencephalography (EEG) to identify abnormal brain activity and often neuroimaging (MRI) to detect structural abnormalities [2]. In clinical practice, epilepsy patients who are candidates for surgical intervention often undergo prolonged EEG monitoring, during which accurate identification of epileptic activity is critical for localizing the epileptogenic zone and guiding surgical decisions. Given the time-intensive nature of manual EEG review, automated detection of epileptic activity is valuable primarily for expediting this process for epileptologists working under considerable time constraints, rather than for exceeding the reliability of expert visual assessment, which remains the diagnostic reference standard.

EEG can capture both ictal (occurring during seizure) and interictal (occurring between seizures) activity, making it indispensable for distinguishing epileptic from non-epileptic events [6]. Interictal activity such as spikes (20–70 ms) and sharp waves (70–200 ms), i.e., Interictal Epileptiform Discharges (IEDs), are used to localize the irritative zone, whereas ictal patterns help identify the seizure onset zone [6,7]. Generalized seizures often exhibit bilaterally synchronous spike-and-wave discharges, while focal seizures are typically associated with localized interictal activity [8]. Accurate detection of IEDs is crucial for diagnosing epilepsy and avoiding misdiagnosis, which occurs in 20% to 30% of referred patients [9,10].

Morphological analysis of IEDs can be quantified using scoring systems, such as the Bergen Epileptiform Morphology Score (BEMS), which weights features like spike slope and amplitude to improve diagnostic accuracy [11]. However, spike detection remains challenging due to low signal-to-noise ratio, variable morphology, and subjectivity in visual annotation, motivating numerous automated algorithms using signal processing and pattern recognition to enhance consistency [12,13].

Given the substantial inter-individual variability in the morphology and spatial distribution of epileptiform activity, patient-specific machine-learning approaches have been proposed for seizure-onset detection, spike-and-wave classification [14,15,16]. Accordingly, the inferential aim of the present study is restricted to the relative methodological effects observed within one well-characterized patient rather than to estimation of population-level classification performance. Sophisticated Machine Learning models were developed, such as Artificial Neural Networks (ANNs), which learn directly from raw EEG signals to improve the accuracy and generalizability of IED detection [17,18]. Chung et al. [19] proposed deep learning-based models to detect focal IEDs in EEG to enhance EEG interpretation. While our work at hand will also focus on non-invasive surface EEG, Partamian et al. [20] proposed AI-based, patient-specific frameworks for intracranial EEG (iEEG) data to transform IEDs into spatiotemporal representations of brain activity, eliminating the need for manual data inspection. In addition to traditional amplitude- and morphology-based measures, recent studies suggest that epileptiform spikes exhibit complex, nonlinear and transient dynamics. Consequently, nonlinear and complexity-based descriptors such as entropy, fractal dimensions and spectral features have been shown to provide discriminative information for automated spike detection [21]. These features capture complementary aspects of spike morphology that may not be apparent in simple amplitude or duration measurements, potentially improving classification performance when combined with machine learning approaches.

EEG data analysis can be conducted in two complementary domains. Signal-space analysis examines the potential differences measured by scalp electrodes that arise from electrical activity in the brain. While straightforward, this approach is affected by volume conduction, which can complicate the interpretation of underlying neural sources [22,23]. In contrast, source-space analysis applies forward and inverse modeling techniques, incorporating a biophysical head model, to estimate the cortical generators of the measured EEG signals and improve spatial interpretability [24,25]. The accuracy of source-space localization for transient events such as IEDs has been examined across a range of localization approaches, electrode densities, and validation strategies against surgical or intracranial ground truth [26,27,28,29,30]. Ossadtchi et al. [31] describe a fully automated method that combines time-series analysis with MEG source localization to detect clusters of focal neuronal current generators within the brain during interictal spike activity. Their work improves upon automated techniques that are merely based on matching of interictal spike templates or predictive filtering of the data without explicitly including source localization as part of the analysis.

Building on these works, the present study investigates whether EEG features derived from source-space representations provide complementary or superior to traditional signal-space features for automated interictal spike detection. We hypothesize that training Artificial Neural Networks on source-space EEG data improves the reliability of spike detection compared to signal-space analysis alone. To test this hypothesis, EEG signals are filtered and annotated; MRI-based head models are constructed and source localization is performed to obtain source waveforms. ANN-based classifiers are then trained and evaluated using features extracted separately from signal-space and source-space representations.

A further key distinguishing aspect of this study is the availability of annotations from three experienced epileptologists who independently reviewed extended EEG recordings. This enables not only the development of automated detection methods but also the comparison of inter-rater variability with the performance of ANN-based approaches.

## 2. Materials and Methods

### 2.1. Ethics Statement

The study was conducted according to the guidelines of the Declaration of Helsinki and approved by the institution’s ethical review boards (Ethik Kommission der Ärztekammer Westfalen-Lippe und der Westfälischen Wilhelms-Universität Münster, 25 May 2021, Ref. No. 2021-290-f-S). The patient gave written informed consent for her data to be used in anonymized form for scientific publications.

### 2.2. Data Collection

EEG data were collected from a 26-year-old female patient with refractory focal epilepsy originating in the left frontal lobe near Broca’s area, treated at Münster University Hospital between 2018 and 2023 [30]. The patient experienced non-motor seizures characterized by impaired thinking and communication, occurring at a frequency ranging weekly to as often as four times daily. Despite multiple anti-seizure medications, the condition remained pharmacoresistant, prompting a presurgical evaluation in 2018. As the initial invasive EEG did not localize the seizure onset zone, a second EEG/MEG-guided invasive evaluation using depth electrodes (iEEG) was performed in autumn 2021. Subsequently, craniotomy and resection under awake conditions were conducted in January 2023. Following surgery, the patient showed no neurological deficits and achieved seizure freedom under continued medication (Engel Class IA / ILAE class 1), with postoperative MRI confirming correct resection localization. Additional clinical details can be found in Section S2.2 and S2.10 of the Supplementary Material in [30].

In total, 22 h of scalp EEG were recorded using a 19-channel 10–20 system at a sampling rate of 200 Hz and segmented into 22 one-hour sessions. All analyses were performed in MATLAB R2025b using FieldTrip (https://www.fieldtriptoolbox.org) [32], the Signal Processing Toolbox (accessed on 2 September 2026) (https://www.mathworks.com/products/signal.html), the Statistics and Machine Learning Toolbox (accessed on 2 September 2026) (https://www.mathworks.com/products/statistics.html) and some custom scripts.

### 2.3. EEG Preprocessing

EEG preprocessing was performed to ensure high signal quality for reliable spike detection. Recordings from 19 clinical channels of the international 10–20 system were analyzed, with particular relevance to the left frontal region given the F3-centered IEDs investigated in this study [33]. All 19 channels were available and reviewed by the epileptologists during annotation; the left frontal bipolar montage was used solely as the preferred display configuration to facilitate identification of phase reversals associated with focal activity, and did not restrict the channels considered during review. For computational analysis, continuous EEG recordings were bandpass filtered between 0.5 and 40 Hz to suppress slow drifts and high-frequency noise. No additional artifact rejection or correction (e.g., ICA-based removal of ocular or muscle artifacts) was applied beyond this bandpass filtering step. The cleaned recordings, totaling 22 h, were segmented into non-overlapping 1-s epochs (200 samples at a sampling rate of 200 Hz), yielding 79,876 epochs for subsequent analysis.

IEDs were manually annotated in the preprocessed EEG by three independent epileptologists (EP1, EP2 and EP3). Each reviewer independently labeled epochs they judged to contain IEDs, resulting in 2890 spike epochs identified by EP1, 1646 by EP2 and 10,279 by EP3. This variability reflects the well-documented subjectivity of visual EEG interpretation. All remaining epochs were labeled as non-spike, leading to pronounced class imbalance with non-spike epochs substantially outnumbering spike epochs.

To examine the impact of labeling strategy and reduce subjectivity, two complementary label sets were constructed. An AND set included only epochs labeled as spikes by all three epileptologists, providing high-certainty spike annotations. An OR set included epochs marked by at least one reviewer, increasing sensitivity to subtle epileptiform activity at the cost of greater label uncertainty. These labeling strategies formed the basis for subsequent signal- and source-space analysis, feature extraction and ANN model training.

### 2.4. Signal Space Representation

In the signal-space representation, EEG data were analyzed directly at the scalp electrode level using the preprocessed recording described above. Each non-overlapping 1-s epoch (200 samples at 200 Hz) from the 19 electrodes of the international 10–20 system was treated as an independent observation. No spatial transformation or source modeling was applied in this representation and the recorded potentials reflect the mixed contributions of underlying cortical generators as measured at the scalp.

Signal-space epochs labeled according to the AND and OR annotation strategies served as the baseline data for feature extraction and ANN-based spike classification. This representation constitutes the conventional approach used in clinical EEG analysis and provides the reference against which the source-space representation was compared in terms of spike detection performance.

### 2.5. Source Localization

We deliberately performed a standard clinical source reconstruction for the 19-channel EEG dipolar IED topographies by fitting a single equivalent current dipole (ECD) in a realistically shaped three-compartment (scalp, skull, brain) head volume conductor model, built from the individual T1-MRI. Spike epochs identified using the AND and OR labeling strategies were averaged separately and temporally aligned to the negative peak at electrode F3, where maximal spike amplitude was observed, in order to obtain representative spike waveforms for source analysis. Non-spike epochs were retained as unaligned 1-s segments.

Grand-average waveforms were then inspected to identify a stable interval for dipole fitting (Figure 1). The early rising phase (−20 to −5 ms relative to the F3 peak) was selected, as it more closely reflects the primary epileptic generator and is less influenced by propagation effects that may dominate at the spike peak [29,30,34,35,36,37].

An individual T1-weighted MRI was used to construct a realistically shaped three-compartment (scalp, skull, brain) head volume conductor model for symmetric Boundary Element Method (BEM) EEG forward modeling implemented via OpenMEEG in FieldTrip [38]. Standard 10–20 electrode positions were aligned to the scalp surface and a 3-mm source grid was defined within the brain volume. The resulting lead field matrix provided the forward mapping between cortical sources and scalp potentials. All steps were visually inspected to ensure anatomical accuracy and spatial consistency prior to inverse modeling [39].

We estimated the noise covariance matrix from the signal-free baseline and, due to the dipolar topography, a single moving equivalent dipole (ECD) was fitted to the grand-average spike waveforms for both labeling strategies within the selected time window [40,41]. Both AND- and OR-labeled averages yielded high goodness-of-fit values, indicating that the modeled dipoles accounted for most of the observed variance. Dipoles were localized consistently to the left frontal lobe near F3, in agreement with the clinically identified seizure onset zone [30].

Based on the dipole location, source nodes were selected in two stages: (1) an anatomically constrained left frontal region for fixed-orientation (1D) projection and (2) a localized patch of ten nodes surrounding the dipole for evaluating the effect of orientation parameterization. For each of these nodes, source waveforms were computed using three orthogonal orientations (3D, yielding 3 orientations × 10 nodes = 30 components) (Figure 2). We then computed the L2-norm (vector magnitude) of the three-component waveform at each node.

### 2.6. Dataset Balancing

Following signal space representation and source space reconstruction, class imbalance between spike and non-spike epochs was addressed prior to feature extraction. Severe class imbalance can bias machine learning models toward the majority class and distort subsequent feature estimation, particularly for rare events such as IEDs [42].

To mitigate this effect, spike and non-spike epochs were randomly down-sampled to create balanced datasets with equal class representation. This procedure was applied separately for AND and OR labeling strategies. The resulting balanced epoch sets were then used as input for subsequent feature extraction in both signal and source space and for ANN model training. This approach ensured unbiased feature distributions and improved model generalization [43,44].

### 2.7. Feature Extraction

To prepare the data for ANN-based epileptic spike detection, feature extraction was applied to both signal-space and source-space EEG representations. In the signal-space representation, features were extracted independently from each of the 19 EEG channel time series. In the source-space representation, the same feature extraction procedure was applied to each reconstructed source-level time series.

For notational simplicity, x(i), i=1,2,…,N, denotes a one-dimensional, epoch-based time series obtained either from an EEG channel in signal space or from a reconstructed source-level waveform within a given 1-s epoch. Since the sampling frequency was $f_{s}\;=\;200$ Hz, each epoch contained N=200 samples. Thus, $x\left(i\right)=x\left(t_{i}\right)$ denotes the signal amplitude at the *i*-th sample, where $t_{i}=\left(i-1\right)/f_{s}$. All feature definitions presented below are, therefore, applicable to both signal-space and source-space representations. Unless otherwise stated, $\bar{x}$ and $s_{x}$ denote the sample mean and sample standard deviation of x(i), respectively.

The extracted features were grouped into three main categories: statistical, nonlinear, and frequency-domain features. Statistical features characterized the amplitude distribution of each epoch; nonlinear features quantified signal complexity, irregularity, and chaotic dynamics and frequency-domain features described the spectral power distribution across conventional EEG frequency bands [45].

#### 2.7.1. Statistical Features

The mean amplitude value is calculated as:

(1) $$\bar{x}=\frac{1}{N}\sum_{i=1}^{N}x\left(i\right).$$

The amplitude standard deviation is defined as:

(2) $$s_{x}=\sqrt{\frac{1}{N-1}\sum_{i=1}^{N}{\left[x\left(i\right)-\bar{x}\right]}^{2}}.$$

Skewness, which represents the asymmetry of the amplitude distribution, is calculated as:

(3) $$\mathrm{Skewness}\left(x\right)=\frac{\frac{1}{N}\sum_{i=1}^{N}{\left[x\left(i\right)-\bar{x}\right]}^{3}}{{\left[\frac{1}{N}\sum_{i=1}^{N}{\left[x\left(i\right)-\bar{x}\right]}^{2}\right]}^{3/2}}.$$

Kurtosis, which indicates the peakedness or outlier tendency of the amplitude distribution, is computed as:

(4) $$\mathrm{Kurtosis}\left(x\right)=\frac{\frac{1}{N}\sum_{i=1}^{N}{\left[x\left(i\right)-\bar{x}\right]}^{4}}{{\left[\frac{1}{N}\sum_{i=1}^{N}{\left[x\left(i\right)-\bar{x}\right]}^{2}\right]}^{2}}.$$

#### 2.7.2. Nonlinear Features

Nonlinear features were extracted to characterize the complexity, irregularity, and chaotic behavior of the EEG time series. In this study, the same nonlinear feature extraction procedure was applied to both signal-space and source-space EEG representations.

##### Higuchi Fractal Dimension

The Higuchi fractal dimension (HFD) is used to quantify the scale-dependent complexity of each EEG time series. The method constructs subsampled sequences at different scale values and estimates the fractal dimension from the relationship between curve length and scale.

For each scale value $k=1,\ldots ,k_{\max}$ and initial index m=1,…,k, a subsampled sequence is constructed by taking every *k*-th sample of x(i) starting at *m*, up to $\left\lfloor \frac{N-m}{k}\right\rfloor$ points, where ⌊·⌋ denotes the floor function. The normalized curve length of this subsampled sequence is calculated as

(5) $$L_{m}\left(k\right)=\frac{N-1}{\left\lfloor \frac{N-m}{k}\right\rfloor k^{2}}\sum_{q=1}^{\left\lfloor (N-m)/k\right\rfloor }\left|x\left(m+qk\right)-x\left(m+(q-1)k\right)\right|.$$

Here, *q* indexes consecutive points within the subsampled sequence.

The average curve length for scale *k* is then obtained by averaging over the *k* possible initial indices:

(6) $$L\left(k\right)=\frac{1}{k}\sum_{m=1}^{k}L_{m}\left(k\right).$$

HFD is estimated from the slope of the least-squares linear regression of logL(k) against log(1/k):

(7) $$\log L\left(k\right)=\beta_{0}+\beta_{1}\log\left(1/k\right),\;k=1,\ldots ,k_{\max}.$$

The Higuchi fractal dimension is defined as the estimated regression slope:

(8) $$\mathrm{HFD}\left(k_{\max}\right)=\beta_{1}.$$

In the present study, $k_{\max}$ was set to 82, following the recommendation of Doyle et al. [46] for selecting an appropriate scale range in fractal dimension estimation, and was kept constant for all epochs.

##### Katz Fractal Dimension

The Katz fractal dimension (KFD) is used to quantify the geometric complexity of each EEG time series.

(9) $$L=\sum_{i=1}^{N-1}\sqrt{1+{\left[x(i+1)-x(i)\right]}^{2}}.$$

Here, *L* denotes the total curve length, and the horizontal distance between two consecutive samples is taken as one sampling interval.

The maximum Euclidean distance between the first point and any subsequent point in the time series is calculated as

(10) $$d=\max_{2\leq i\leq N}\sqrt{{\left(i-1\right)}^{2}+{\left[x(i)-x(1)\right]}^{2}}.$$

Here, *d* denotes the maximum extent of the curve relative to its first point.

The average distance between consecutive points is defined as

(11) $$a=\frac{L}{N-1}.$$

Here, *a* denotes the average step length along the curve.

(12) $$\mathrm{KFD}\left(x\right)=\frac{\log(L/a)}{\log(d/a)}.$$

Larger KFD values indicate greater geometric complexity of the time series. While Katz and Higuchi fractal dimensions capture the geometric complexity of the EEG waveform and proved to be the most discriminative features in this study (see Results), we additionally examined three complementary nonlinear measures (the largest Lyapunov exponent, approximate entropy, and Shannon entropy) to test whether chaotic-dynamics and information-theoretic descriptors provide further discriminative value beyond fractal-dimension-based features.

##### Lyapunov Exponent

The largest Lyapunov exponent characterizes the average rate at which initially neighboring trajectories diverge in the reconstructed state space, with positive values indicating chaotic dynamics. State-space reconstruction uses an embedding dimension $m_{e}$ and time delay τ, and the exponent is estimated from the average exponential divergence rate between neighboring reconstructed states, following Rosenstein et al. [47]. In this study, the largest Lyapunov exponent was computed for each epoch and channel using the *lyapunovExponent* function of MATLAB’s Predictive Maintenance Toolbox (accessed on 10 November 2024) (https://www.mathworks.com/help/predmaint/ref/lyapunovexponent.html), which implements the algorithm of Rosenstein et al. [47]. The embedding dimension and time delay were not fixed a priori but were determined automatically per epoch, using the false nearest neighbors method for $m_{e}$ and the first minimum of the average mutual information for τ.

##### Approximate Entropy

Approximate entropy (ApEn) quantifies the regularity and predictability of each EEG time series, with lower values indicating more regular patterns. ApEn is computed by comparing *m*-length and (m+1)-length template vectors of consecutive samples and measuring the log-difference in the proportion of vectors that remain similar within a tolerance *r*, following Pincus [48]. In the present study, m=2 and $r=0.2\;s_{x}$, as recommended by Pincus [48].

##### Shannon Entropy

Shannon entropy is used to quantify the uncertainty and energy concentration of each EEG time series based on its amplitude values, with higher values indicating a more irregular and less concentrated signal. Following the implementation of the *wentropy* function in MATLAB’s Wavelet Toolbox (accessed on 10 November 2024) (https://www.mathworks.com/help/wavelet/ref/wentropy.html) (‘shannon’ entropy type), only strictly positive amplitude values contribute to the entropy calculation.

#### 2.7.3. Frequency-Domain Features

Frequency-domain features characterize the spectral power distribution of each epoch-based EEG time series. The power spectral density (PSD) of each epoch-based time series was estimated using Welch’s method [49], with a 100-sample Hamming window, 50% overlap between consecutive segments, and a 256-point FFT. The signal is divided into overlapping windowed segments, the periodogram of each segment is computed, and the resulting periodograms are averaged to obtain the PSD. Band power was then obtained by integrating the PSD within the conventional EEG frequency bands: delta (0.5–4 Hz), theta (4–8 Hz), alpha (8–13 Hz), beta (13–30 Hz), and gamma (30–60 Hz). The resulting delta, theta, alpha, beta, and gamma band power values are used as frequency-domain features. The same procedure is applied independently to each EEG channel in signal space and to each reconstructed source-level time series in source space.

### 2.8. Artificial Neural Networks

Artificial Neural Networks (ANNs) are computational models inspired by the biological nervous system, in which simplified artificial neurons compute weighted sums of inputs, apply a nonlinear activation function, and generate outputs [50]. A feed-forward ANN with one hidden layer of 10 neurons and a single output neuron was used in this study for binary spike classification. The number of hidden neurons was not optimized via a formal hyperparameter search; instead, this architecture was deliberately kept simple and fixed across all signal-space and source-space representations, so that observed performance differences could be attributed to the input representation and extracted features rather than to architectural differences [51].

For each input feature vector $x=[x_{1},x_{2},\ldots ,x_{p}]$, the input to the *j*-th hidden neuron is calculated as:

(13) $$z_{j}=\sum_{i=1}^{p}w_{ji}x_{i}+b_{j},$$

where $w_{ji}$ is the connection weight between the *i*-th input feature and the *j*-th hidden neuron, $b_{j}$ is the bias term, and *p* is the number of input features. Both $w_{ji}$ and $b_{j}$ are learned during training. The hidden-layer output is obtained as:

(14) $$h_{j}=f\left(z_{j}\right),$$

where f(·) is the hyperbolic tangent sigmoid (tansig) activation function.

The network output is then computed as:

(15) $$\hat{y}=g\left(\sum_{j=1}^{H}v_{j}h_{j}+c\right),$$

where H=10 is the number of hidden neurons, $v_{j}$ is the output-layer weight, *c* is the output bias, both likewise learned during training, and g(·) is the softmax activation function, so that $\hat{y}$ represents the predicted probability of the spike class.

Separate feed-forward ANN models with identical architectures were trained using MATLAB’s *patternnet* (accessed on 13 November 2024) (https://www.mathworks.com/help/deeplearning/ref/patternnet.html). By default, training was performed using the Scaled Conjugate Gradient (SCG) backpropagation algorithm [52], which efficiently incorporates second-order curvature information without explicit Hessian computation or line search. The network was trained by minimizing cross-entropy loss between predicted and true class labels.

All input features were z-score normalized prior to training to ensure comparable scaling across features:

(16) $$x_{i}^{norm}=\frac{x_{i}-\mu_{i}}{\sigma_{i}},$$

where $\mu_{i}$ and $\sigma_{i}$ denote the mean and standard deviation of the *i*-th feature, respectively. To avoid data leakage, normalization parameters were estimated from the training data within each cross-validation fold and then applied to the corresponding test data.

**Signal-space model:** The raw input representation used 3800-dimensional vectors (19 channels × 200 time points) from average-referenced EEG epochs.

**Source-space model:** For the 1-parameter projection, the input consisted of 2000-dimensional vectors (10 nodes × 200 time points). For the 3-parameter projection, the input consisted of 6000-dimensional vectors (30 components × 200 time points), preserving the three orthogonal current directions at each node.

Model performance was evaluated through 10-fold cross-validation to ensure robust generalization and prevent overfitting [53,54]. Within each fold, the training data were further subdivided (70% training, 30% testing) for internal model selection. Classification performance was evaluated using multiple complementary metrics, including accuracy, sensitivity, specificity, precision, F1-score, geometric mean (G-mean), and Cohen’s Kappa. Accuracy measures overall correctness, while sensitivity and specificity reflect the correct identification of spikes and non-spike predictions and F1-score balances precision and recall [55,56]. The G-mean assesses the balance between sensitivity and specificity, making it useful for imbalanced datasets. Cohen’s Kappa evaluates agreement beyond chance, with values above 0.6 indicating substantial agreement [57,58].

## 3. Results

This section presents the classification performance of the ANN under different data representations and preprocessing strategies, with the primary goals of (i) comparing signal and source space IED detections, the latter with (ii) fixed and free source orientations, (iii) evaluating the impact of feature extraction on both as well as (iv) using AND and OR subsets of epochs and evaluating the quality of our automatic procedures in the light of the inter-rater differences.

All performance metrics reported below should, therefore, be interpreted as within-patient estimates, with emphasis placed on relative differences between methodological conditions rather than on absolute performance as an estimate of external validity.

ANN performance was assessed for four conditions: raw signal-space EEG, feature-extracted signal-space EEG, raw source-space representation and feature-extracted source-space data. Results are reported separately for AND- and OR-marking strategies to account for differences in annotation certainty. Baseline performance using raw, non-feature-extracted data across all four conditions is summarized in Table 1. Feature-extracted performance was evaluated in Table 2, Table 3 and Table 4 systematically across individual features (F1–F5, F10, F12) and their combinations (F6–F9, F11), this comparison also functions as a feature-importance analysis, allowing the relative contribution of each feature type to classification an accuracy to be assessed directly from the Tables.

### 3.1. Signal-Space Classification

Using raw signal-space EEG epochs without feature extraction resulted in near-chance classification performance for both marking strategies (Table 1). Accuracies were 0.52 (AND) and 0.51 (OR), barely exceeding random guessing. Cohen’s kappa values were low, indicating agreement only slightly better than chance [57]. Sensitivity and specificity were unbalanced, with neither metric consistently exceeding 0.60, demonstrating that raw average-referenced EEG waveforms alone are insufficient for reliable ANN-based IED detection.

In contrast, feature extraction led to a considerable improvement in classification performance (Table 2). For AND-marked data, several feature sets achieved accuracies above 0.97, with balanced sensitivity and specificity exceeding 0.95, indicating balanced performance across both spike and non-spike classes. Most notably, the Katz Fractal Dimension (KFD, F2) alone reached accuracy of 0.98 (Cohen’s κ=0.96, indicating almost perfect agreement), comparable to more complex combined feature sets such as F6 (Statistical + KFD + Power Spectral Density (PSD): 0.98), F8 (KFD + Lyapunov: 0.98) and F11 (KFD + Approximate Entropy: 0.97). Notably, sensitivity and specificity for this best-performing model both exceeded 0.97, indicating balanced performance with low rates of both missed spikes and false alarms—a consideration of particular relevance for presurgical evaluation, where missed epileptiform activity could affect diagnosis and/or localization of the epileptogenic zone.

Performance for OR-marked data was lower overall (best accuracy: 0.90) but still substantially improved compared to raw signal space (0.51). Best-performing feature sets achieved accuracies between 0.88 and 0.90, with F2 again leading with an 0.88 accuracy, closely followed by F6, F7 and F9 (Statistical + KFD). Cohen’s kappa values ranged from 0.76 to 0.80, indicating substantial agreement. The systematically lower performance for OR-marking reflects the increased morphology variability and subjectivity inherent in this labeling scheme, where spikes are marked by at least one epileptologist.

These results demonstrate that appropriate feature engineering transforms near-random performance (0.51–0.52) into consistently high classification accuracy (0.88–0.98). Notably, in both results and those reported by [59], KFD alone achieved performance levels close to the best feature combinations, suggesting that KFD is the primary driver of high accuracy, while combinations offer only marginal additional benefit.

**Table 1:** ANN Classification Performance for Raw EEG and Source-space Data Across AND- and OR-marking Strategies.

Metric	Signal Space		Source Space (Fixed)		Source Space (Free)	
	AND	OR	AND	OR	AND	OR
Accuracy	0.521	0.512	0.5375	0.5116	0.5958	0.5872
Sensitivity	0.428	0.498	0.6002	0.6614	0.5799	0.6263
Specificity	0.613	0.525	0.4748	0.3617	0.6116	0.5480
Precision	0.526	0.512	0.5333	0.5089	0.5989	0.5809
F1-score	0.472	0.505	0.5648	0.5752	0.5892	0.6027
G-Mean	0.513	0.511	0.5338	0.4891	0.5956	0.5859
Cohen’s Kappa	0.042	0.024	0.5374	0.5115	0.1915	0.1744

**Table 2:** Signal Space ANN Classification Performance for AND- and OR-marking.

Feature	AND-Marking			OR-Marking		
	Accuracy	Sensitivity	Specificity	Accuracy	Sensitivity	Specificity
F1 (Statistical)	0.94	0.95	0.94	0.84	0.87	0.81
F2 (KFD)	0.98	0.99	0.97	0.88	0.94	0.83
F3 (Higuchi FD)	0.87	0.88	0.86	0.68	0.66	0.70
F4 (Lyapunov)	0.91	0.92	0.90	0.77	0.79	0.76
F5a (PSD, γ, β)	0.85	0.86	0.85	0.74	0.77	0.72
F5b (PSD, all bands)	0.94	0.96	0.93	0.69	0.51	0.88
F5c (PSD, α, δ, θ)	0.88	0.91	0.84	0.62	0.33	0.91
F6 (F5a + F2 + F1)	0.98	0.98	0.97	0.89	0.92	0.86
F7 (F5a + F2 + F1 +F4)	0.97	0.98	0.97	0.90	0.93	0.87
F8 (F2 + F4)	0.98	0.98	0.97	0.89	0.94	0.83
F9 (F1 + F2)	0.97	0.97	0.97	0.90	0.93	0.88
F10 (Approximate Ent.)	0.92	0.93	0.92	0.80	0.80	0.80
F11 (F2 + F10)	0.97	0.97	0.97	0.89	0.94	0.85
F12 (Shannon Ent.)	0.87	0.89	0.85	0.67	0.51	0.83

### 3.2. Source-Space Classification

Applying ANN classification directly to source-localized waveforms without feature extraction yielded only modest improvements over raw signal space (Table 1). The fixed-orientation model achieved accuracy of 0.53 (AND) and 0.51 (OR), barely above chance. The free-orientation model achieved slightly higher accuracies of 0.59 (AND) and 0.58 (OR). However, performance remained substantially lower than that achieved using feature-extracted signal-space data, indicating that the dimensionality reduction by source analysis alone does not improve the classification much.

Feature extraction in source space improved classification performance relative to raw source data (Table 3 and Table 4), but peak accuracies remained lower than those obtained in signal space. For the fixed-orientation model with AND-marked data, the maximum accuracy of 0.84 was achieved with statistical features (F1) and mixed feature sets performing best (F6, F7). For the free-orientation (3-parameter) model, best performance increased to 0.85 (F6, F7) and F2 being 0.84, representing only a modest improvement despite preserving full three-dimensional source orientation information.

**Table 3:** Source Space ANN Classification Performance for Fixed-Orientation Model (AND- and OR-marking).

Feature	AND-Marking			OR-Marking		
	Accuracy	Sensitivity	Specificity	Accuracy	Sensitivity	Specificity
F1 (Statistical)	0.84	0.81	0.86	0.65	0.55	0.74
F2 (KFD)	0.68	0.62	0.73	0.59	0.59	0.60
F3 (Higuchi FD)	0.62	0.72	0.51	0.59	0.67	0.52
F4 (Lyapunov)	0.59	0.65	0.52	0.57	0.60	0.53
F5a (PSD, γ, β )	0.77	0.67	0.86	0.50	0.37	0.63
F5b (PSD, all bands)	0.78	0.72	0.85	0.50	0.65	0.36
F5c (PSD, α, δ, θ)	0.59	0.75	0.43	0.51	0.64	0.37
F6 (F5a + F2 + F1)	0.84	0.81	0.87	0.64	0.59	0.70
F7 (F5a + F2 + F1 +F4)	0.84	0.83	0.85	0.65	0.59	0.71
F8 (F2 + F4)	0.66	0.66	0.66	0.58	0.58	0.59
F9 (F1 + F2)	0.83	0.82	0.85	0.65	0.57	0.73
F10 (Approximate Ent.)	0.61	0.63	0.59	0.56	0.62	0.50
F11 (F2 + F10)	0.69	0.69	0.68	0.58	0.61	0.55
F12 (Shannon Ent.)	0.63	0.80	0.45	0.53	0.88	0.16

**Table 4:** Source Space ANN Classification Performance for Free-Orientation Model (AND- and OR-marking).

Feature	AND-Marking			OR-Marking		
	Accuracy	Sensitivity	Specificity	Accuracy	Sensitivity	Specificity
F1 (Statistical)	0.75	0.73	0.77	0.64	0.59	0.69
F2 (KFD)	0.84	0.84	0.84	0.68	0.59	0.69
F3 (Higuchi FD)	0.63	0.68	0.58	0.61	0.67	0.56
F4 (Lyapunov)	0.63	0.70	0.57	0.60	0.63	0.57
F5a (PSD, γ, β )	0.80	0.75	0.85	0.54	0.80	0.28
F5b (PSD, all bands)	0.82	0.76	0.87	0.54	0.84	0.25
F5c (PSD, α, δ, θ)	0.53	0.90	0.25	0.53	0.90	0.16
F6 (F5a + F2 + F1)	0.85	0.84	0.86	0.65	0.60	0.69
F7 (F5a + F2 + F1 +F4)	0.85	0.84	0.86	0.67	0.64	0.70
F8 (F2 + F4)	0.82	0.81	0.82	0.63	0.65	0.61
F9 (F1 + F2)	0.73	0.71	0.75	0.60	0.58	0.63
F10 (Approximate Ent.)	0.65	0.68	0.63	0.60	0.62	0.58
F11 (F2 + F10)	0.83	0.72	0.83	0.64	0.66	0.61
F12 (Shannon Ent.)	0.68	0.78	0.58	0.53	0.83	0.22

Notably, KFD (F2), which achieved 0.98 accuracy in signal space, showed substantially reduced performance in source space: 0.68 (1D) and 0.84 (3D) for AND marking. This suggests that the source projection, while improving spatial specificity, may compress spatiotemporal signal complexity critical for certain nonlinear features. The free-orientation model partially recovered performance (0.84 vs. 0.68), supporting the importance of preserving orientation dynamics for complexity-based features, though the improvement remained modest compared to the signal-space benchmark.

For OR-marked data, overall performance was further reduced, with best accuracies around 0.65 (fixed) and 0.68 (free), compared to 0.90 in signal space. This decline highlights the sensitivity of source-space feature-based classification to labeling uncertainty and increased variability.

Overall, feature extraction was the dominant factor in improving classification in both signal and source spaces (from 0.52 to 0.84–0.98), but signal-space features outperformed source-space features. While source localization offers improved spatial interpretability, it did not enhance classification performance. The free-orientation model provided only marginal gains over fixed orientation (0.85 vs. 0.84 for AND-marking).

We note that cross-validation was performed at the epoch level; because temporally adjacent epochs from the same continuous recording could, therefore, appear in both training and test folds within a fold, the accuracies reported above may be modestly optimistic relative to a stricter recording- or block-wise validation scheme. This methodological consideration is discussed further, together with its implications for future work, in Section 4.

## 4. Discussion

This is the first study where 22 h of continuous EEG data, independently marked by three experienced epileptologists, were used to systematically train and evaluate Artificial Neural Networks with and without feature extraction in signal space and source space. By comparing raw waveform classification against feature-based approaches across multiple representation domains (signal space, 1-parameter source projection and 3-parameter source projection), we provide a comprehensive assessment of the methodological factors that determine automated spike detection performance. Our proof-of-principle case study results demonstrate that feature extraction may be important for reliable ANN-based IED detection, whereas raw EEG data in both signal and source space may lack sufficient discriminative structure for accurate classification without dimensionality reduction through carefully designed features.

In signal space, the application of morphological, spectral and nonlinear features significantly improved performance, achieving accuracies up to 0.98 for AND-marked data and 0.90 for OR-marked data. Raw signal-space waveforms, by contrast, achieved only chance-level performance (accuracy ≈ 0.52), suggesting that the high-dimensional EEG time series used here did not provide sufficient discriminative structure for the conventional classifier without feature engineering.

While accuracy provides an overall summary of classification performance, sensitivity and specificity are of particular clinical relevance in this context: a missed IED (false negative) may lead to an incomplete or inaccurate delineation of the irritative zone during presurgical evaluation, whereas a false positive primarily adds a modest reviewing burden for the epileptologist rather than a diagnostic risk. For this reason, the near-balanced and consistently high sensitivity and specificity achieved by the best-performing signal-space model (KFD, AND-marking: sensitivity 0.99, specificity 0.97; Table 2) are at least as clinically meaningful as its accuracy, since they indicate a low rate of missed epileptiform events alongside a low false-alarm rate.

Katz Fractal Dimension (KFD) emerged as the most discriminative single feature, achieving 0.98 accuracy (AND marking) and 0.88 accuracy (OR-marking) without being combined with other features. This finding is consistent with the pioneering work of [59], who demonstrated that KFD achieves near optimal classification performance (0.96 AND-marking, 0.87 OR-marking) when applied to the 12 most informative bipolar channels selected from a standard 19-electrode montage. Our results extend these findings by showing that KFD maintains its discriminative power even when applied to average-referenced data across all 19 channels, achieving comparable performance, which suggests that the choice of referencing scheme may have limited impact on KFD’s discriminative ability once appropriate features are extracted.

By contrast, other individual nonlinear features such as Higuchi FD (0.87 accuracy) and Shannon entropy (0.87 accuracy) showed consistently lower discriminative power than KFD (0.98), indicating that the observed classification gains are driven predominantly by KFD rather than by nonlinear complexity measures more broadly.

The strong performance of fractal dimension measures for spike detection aligns with broader literature on nonlinear EEG analysis. Arle and Simon [60] showed that fractal dimension could distinguish transient epileptiform events from background EEG based on self similar temporal structure. Accardo et al. [61] showed that Higuchi and Katz fractal dimensions effectively quantify the complexity changes associated with pathological brain states. Our finding that KFD substantially outperforms Higuchi FD (0.98 vs. 0.87 accuracy for AND marking) is consistent with Anisheh and Hasanpour [62], who noted that KFD requires less computational complexity while maintaining discriminative power for abrupt waveform changes characteristic of epileptic spikes.

Statistical features (mean, standard deviation, skewness, kurtosis) also achieved strong performance (0.94 accuracy for AND-marking), consistent with Shoka et al. [63], who identified these features among the optimal extraction methods for epilepsy classification across multiple studies. The combination of KFD with statistical features (F9) achieved 0.97 accuracy, demonstrating complementarity between fractal and moment-based descriptors-fractal dimension captures self-similar temporal structure, while statistical moments quantify amplitude distribution properties.

Spectral features based on conventional EEG sub-bands (delta, theta, alpha, beta, gamma) showed variable performance depending on band selection. Beta and gamma bands (F5a: 0.85 accuracy) outperformed all five bands combined (F5b: 0.94), suggesting that higher-frequency components contain spike-specific information, consistent with the known spectral signature of sharp transients. These findings align with Hassan et al. [64], who reported that higher-frequency sub-bands yielded better epileptic seizure detection performance using multi-band feature extraction with feed-forward neural networks.

Entropy-based measures (approximate entropy: 0.92, Shannon entropy: 0.87) showed moderate discriminative power, though they underperformed relative to fractal dimension. This contrasts with some prior work [65] that reported excellent seizure detection using entropy features combined with general regression neural networks. The discrepancy may reflect differences between interictal spike detection and ictal seizure detection, as seizures involve more pronounced state changes that entropy measures are designed to quantify.

The comparison between AND-marking and OR-marking strategies revealed that data consistency plays a critical role in model performance, which may reflect the well-documented variability and subjectivity of visual EEG interpretation reported in literature [66]. AND-marked data, representing events unanimously identified by all three epileptologists, yielded substantially higher classification accuracies (up to 0.98) compared to OR-marked data (up to 0.90). OR-marked data, which includes events identified by at least one expert, introduced greater variability due to its inclusion of ambiguous and heterogeneous spike morphologies. Nevertheless, even under this noisier labeling condition, feature-based representations remained superior to raw input, emphasizing the robustness of feature extraction.

We opted against the Synthetic Minority Over-sampling Technique (SMOTE) [44] and cost-sensitive learning in the present study because our down-sampled datasets remained large (spike-epoch counts ranging from 1646 to 10,279 per labeling strategy), providing ample data for stable ANN training even after balancing, and because random down-sampling preserves genuine EEG waveform morphology rather than introducing synthetic interpolated samples, which is particularly relevant when the discriminative features (e.g., Katz Fractal Dimension) are sensitive to fine-grained signal morphology. A comparison with SMOTE and cost-sensitive alternatives is, however, an interesting direction for future methodological refinement.

Source-space analysis provided insight into the trade-off between anatomical specificity and signal information preservation. Raw source-projected data showed only modest improvements over raw signal-space data (accuracies 0.54–0.60), suggesting that dimensionality reduction through projection alone is insufficient. When features were applied, source-space performance improved substantially, reaching accuracies up to 0.85 (3D projection, AND-marking). However, source-space results did not surpass those obtained in signal space (0.98), indicating that, even for dipolar IED topographies, the deliberately chosen standard clinical source analysis approach of a single equivalent current dipole (ECD) in a three-compartment head model may not preserve all of the discriminative information available in the original signal-space representation. This motivates future investigations using high-density EEG/MEG and improved source analysis approaches regarding both the forward and inverse problem [23,30].

A key finding was the dependence of feature effectiveness on data representation. While KFD dominated in signal space, statistical features became more effective in the 1-parameter source projection. This shift can be explained by the smoothing and noise-suppressing effect of source localization, which produces simpler, more “peaky” waveforms that are well captured by basic statistical measures but less suitable for complexity-based features consistent with previous reports noting that inverse source reconstruction involves spatial regularization and smoothing, which may attenuate fine temporal details of scalp EEG activity [67]. The improved performance of KFD under the free-orientation (3-parameter) source model supports this interpretation (F2: 0.84 vs. 0.68 for 1-parameter), as additional degrees of freedom preserve more dynamic information, such as waveform rotation and propagation from sulcal to gyral regions, as reconstructed in our patient from high-density EEG/MEG recordings [30].

These findings align with previous work showing that feature extraction is fundamental for IED detection and that more complex source models might better capture epileptiform dynamics [68,69]. Unlike studies using distributed source models [70], the simplified projections used here restrict the representational power of the source-space. This limitation explains why source-space analysis, despite its anatomical advantages, did not outperform signal-space analysis in classification accuracy.

The most significant limitation of this study is the single-patient design. While 22 h of continuous EEG provide substantial within-patient variability, epileptic spikes vary considerably across individuals depending on epilepsy etiology, cortical origin, and medication status [7]. Although patient-specific machine-learning approaches can capture individualized epileptiform patterns, they still require validation across multiple patients to establish robustness and external validity. Previous studies have evaluated patient-specific methods across cohorts, including 36 patients in [15] and 43 patients in [20]. In the present study, the 22-h recording enabled detailed within-patient comparisons; however, the large number of epochs does not substitute for inter-patient variability. Therefore, the present findings should be interpreted as a proof-of-principle demonstration of feature-based ANN spike detection within a single, well-characterized dataset, rather than as evidence of generalizable performance across the broader epilepsy population. Multi-patient validation is essential before any claims of clinical generalizability can be made, as previous studies show performance degradation when moving from single-patient to multi-patient scenarios [71,72]. A systematic extension of this work should include patients with varying seizure foci (frontal, temporal, and other origins), source depths, and dipole orientations, in order to evaluate whether the discriminative advantage of Katz Fractal Dimension observed here generalizes across heterogeneous spike morphologies.

Relatedly, the present study did not benchmark the proposed ANN against clinically established spike-detection methods or state-of-the-art deep learning models, which limits conclusions regarding its comparative performance advantage. The feed-forward ANN architecture was intentionally kept simple to allow a controlled, interpretable comparison between signal-space and source-space representations [51]. While this facilitated the present analysis, more advanced architectures, particularly convolutional neural networks (CNNs), have shown promise for capturing spatiotemporal structure in EEG data [72] and represent a natural next step. We did not implement CNN or deep-learning model comparisons in the present study, as doing so would require substantially expanding the scope of the analysis across both signal- and source-space representations without altering the already high classification accuracy (up to 0.98) achieved with the current feature-based approach; we, therefore, identify such a systematic comparison, evaluated on an expanded multi-patient dataset, as a priority for future work.

The 19-channel EEG montage used in this study also constrains the spatial resolution available for source reconstruction, which may bias or destabilize the localization of focal spike generators; this limitation is compounded by our use of a single moving dipole rather than a distributed source model [70]. Connectivity-based approaches applied to source-reconstructed interictal activity, such as Duma et al. [73], who characterized dynamic functional connectivity patterns of source-reconstructed interictal spikes, offer a complementary strategy for contextualizing source-space findings under limited spatial sampling and represent a useful direction for future extensions of this work.

A related methodological consideration concerns the use of grand-averaged waveforms for dipole fitting and source-waveform extraction. This approach assumes that spikes contributing to the average originate from a single, stable underlying generator; if the true epileptiform activity instead arose from multiple distinct or drifting sources, grand-averaging could distort the resulting source estimate. In the present dataset, we found a consistent localization of the fitted dipole to the left frontal region near F3 across both AND- and OR-marking strategies, in agreement with the independently established, clinically confirmed seizure onset zone. Nonetheless, we cannot exclude generator heterogeneity within this region. In [29], we developed a sub-averaging source reconstruction approach, balancing the number of averaged IEDs, and, thus, the resulting SNR, between (i) large enough to avoid systematic error, especially in source depth, due to excessive residual data noise and (ii) as small as possible to avoid losing the extent information of the underlying source area contained in the individual spikes, as would result from global averaging of all IEDs. This subaveraging approach has also been used in [30] for the focal epilepsy patient at hand within the targeting session using high-density 74 channel EEG in combination with whole-head 275 axial gradiometers MEG and a realistic calibrated anisotropic finite element head model built from multimodal MRI data. The main results were that a neighboring sulcus close to Broca was localized underlying clearly dipolar EEG and MEG topographies with no contribution of distant sources, with a propagation from the sulcal valley at spike onset to the gyral crown at spike peak. While one important goal of [30] was to accurately localize the centroid and spread sphere of the patient’s irritative zone for optimal targeting, in the study at hand, we explicitly planned to use a standard clinical localization procedure for the dipolar IED topographies, based only on the available 19-channel EEG and the patient’s T1-MRI. However, we plan to further investigate the effect of an improved source reconstruction with high-density EEG/MEG recordings and multimodal MRI on automatic source space IED detection. Methodologically, the moving dipole fit in the three-compartment head model could be improved using e.g. hierarchical Bayesian modeling [70] or beamforming [74] in a calibrated anisotropic six-compartment head model [23,30,75], which may better preserve discriminative source-space information than the simplified projection approach used here. Furthermore, especially for multifocal epilepsy, connectivity-based approaches such as the generalized partial directed coherence [76] or the time resolved phase locking value [73] might contribute to an improved reconstruction of the underlying epileptic network and represent a useful direction for future extensions of this work.

We also note that model evaluation relied on epoch-level 10-fold cross-validation, in which temporally adjacent segments from the same continuous recording could appear in both training and test folds. This may introduce a degree of temporal dependence between the two sets and could inflate performance estimates relative to a stricter recording- or block-wise cross-validation scheme, in which entire continuous recording blocks are held out from training [77,78]. We did not implement block-wise cross-validation in the present study, and the reported accuracies should, therefore, be interpreted with this caveat in mind. Adopting a recording-wise cross-validation strategy is a priority for the planned multi-patient follow-up study, where a larger number of independent recordings will make such a split feasible without excessively reducing training data.

Finally, clinical validation studies are essential to evaluate whether automated spike detection reduces epileptologist workload while maintaining diagnostic accuracy in real-world clinical workflows.

## 5. Conclusions

This proof-of-principle single case study investigated ANN-based IED detection using EEG data in both signal and source space, with and without feature extraction. The results indicate that feature extraction might contribute considerably to accurate classification, whereas raw EEG input alone might be insufficient. In signal space, the feature-based model achieved the highest performance, reaching accuracies of up to 0.98, with Katz Fractal Dimension and combined spectral-fractal features proving especially effective.

Although source localization provides valuable anatomical information, its integration through a standard clinical approach of low-density EEG in combination with a dipole fit in a three-compartment head model offered only modest gains and did not outperform signal-space performance. The dependence of feature effectiveness on data representation highlights the importance of matching feature design to the underlying signal model. In particular, high-density recordings in combination with more complex source analysis approaches might help preserve the spatiotemporal dynamics of IEDs and better exploit the potential of source-space analysis.

These findings reinforce the critical role of data representation and feature selection in EEG-based IED detection. From a clinical perspective, feature-based automated systems operating in signal space may serve as assistive tools for improving efficiency and consistency in epilepsy diagnostics. Future work should extend this framework to multi-patient datasets, explore richer and more flexible source localization strategies, and investigate CNN or deep learning architectures capable of directly learning spatiotemporal patterns from both signal- and source-space data.

## Figures and Tables

**Figure 1:**
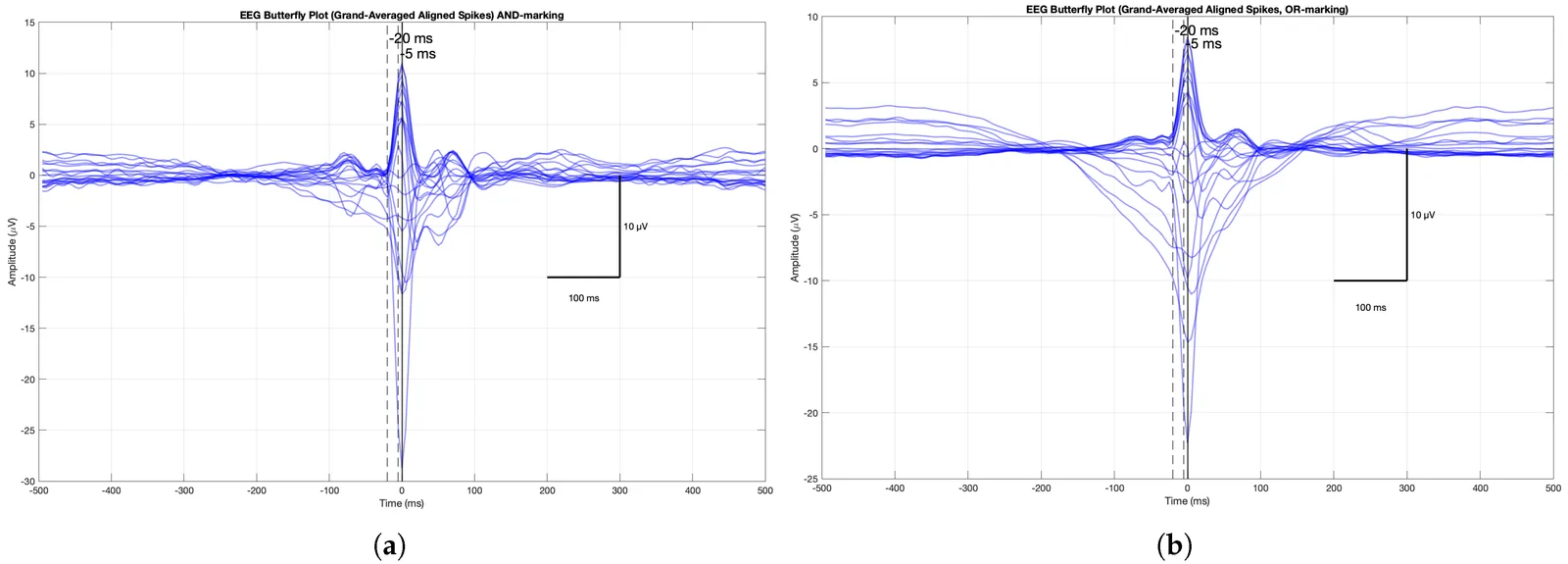
Grand average of IED epochs for (**a**) conservative AND-marking (unanimous expert agreement) and (**b**) sensitive OR-marking (at least one expert) strategies. Each colored trace represents one of 19 EEG channels in average reference. Waveforms are temporally aligned to the negative peak amplitude at the F3 electrode (time = 0 ms, vertical solid line). The vertical dashed lines at −20 ms and −5 ms indicate the time window used for subsequent dipole fitting and source reconstruction. Time axis: −100 ms to +100 ms relative to peak. Amplitude axis: μV.

**Figure 2:**
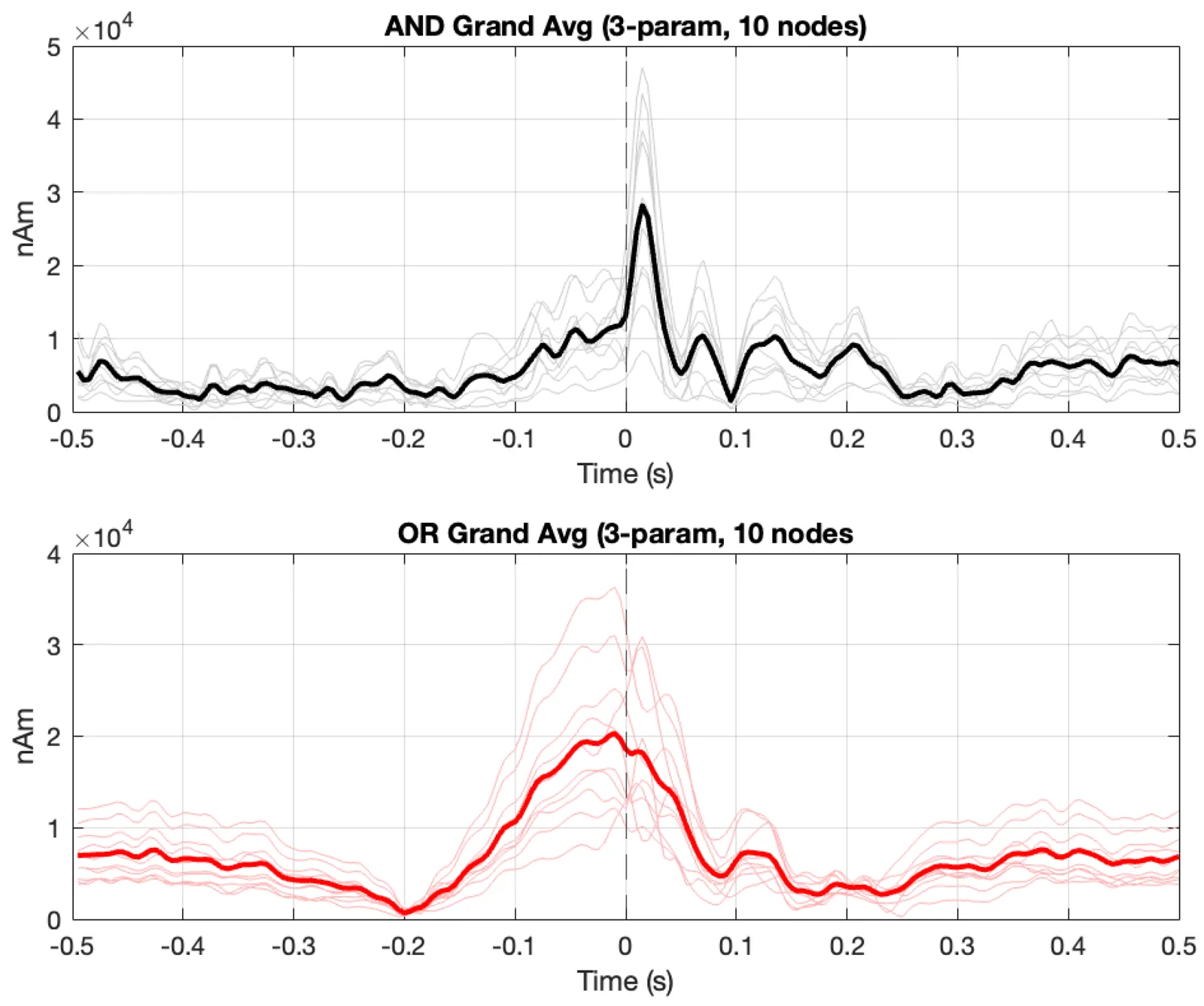
Patch-level source waveforms for grand-average IEDs. The plots show the vector magnitude of the three-component source estimates at each node, expressed in nanoampere-meters (nAm), averaged across the 10 closest cortical nodes to the fitted dipole location for (**a**) AND-marked and (**b**) OR-marked data. Gray lines: individual node magnitudes; black line (**a**)/red line (**b**): mean across nodes.

## Data Availability

All analysis code and processing pipelines are publicly available on GitHub at https://github.com/cafaroval/ML_spike_detection (accessed on 23 July 2025) and data is available in sufficiently anonymized form upon request.
